# Prescribing Trends, Adherence, and Tolerability of Antianxiety Medications in an Indian Tertiary Care Psychiatry Outpatient Department: A Cross-Sectional Study

**DOI:** 10.7759/cureus.115592

**Published:** 2026-09-01

**Authors:** Nisha Sejwal, Amit Kumar Sharma, Deepak Pandey, Sparsh Gupta, Shaily Mina

**Affiliations:** 1 Pharmacology, Vardhman Mahavir Medical College and Safdarjung Hospital, New Delhi, IND; 2 Psychiatry, Vardhman Mahavir Medical College and Safdarjung Hospital, New Delhi, IND

**Keywords:** adverse drug reactions, anxiety disorders, benzodiazepines, medication adherence, polypharmacy, prescribing patterns, ssris

## Abstract

Background

Pharmacological therapy, particularly the use of selective serotonin reuptake inhibitors (SSRIs), plays a central role in managing anxiety spectrum disorders. However, real-world clinical practice can deviate from evidence-based recommendations, leading to challenges such as prolonged benzodiazepine use, polypharmacy, and low rates of generic prescribing. Evaluating these real-world prescribing patterns, alongside patient adherence and adverse drug reactions (ADRs), is crucial for optimizing treatment. This study aimed to describe the prescribing patterns of anti-anxiety medications in a tertiary-care psychiatry outpatient department and to evaluate patient adherence and the incidence of ADRs.

Methodology

A cross-sectional observational study was conducted over six months, enrolling 150 consecutive adult patients (aged 18 to 60 years) diagnosed with anxiety or related disorders based on the International Classification of Diseases, 10th Revision (ICD-10) criteria. Sociodemographic details, clinical diagnoses, and complete prescription data were recorded. Medications were categorized by drug class, with polytherapy defined as the concurrent use of two or more anxiolytic agents. Medication adherence was assessed using the 10-item Medication Adherence Rating Scale (MARS-10). ADRs were documented through targeted patient interviews based on a symptom checklist.

Results

The study cohort had a mean age of 37.6±11.2 years and was predominantly made up of women (62%). Generalized anxiety disorder was the most frequent primary diagnosis (30%), followed by panic disorder (14.7%) and obsessive-compulsive disorder (13.3%). Selective serotonin reuptake inhibitors (SSRIs) were the most widely prescribed medication class, utilized in 73.3% of patients, with sertraline (22.7%) and escitalopram (22.7%) being the preferred agents. Benzodiazepines were prescribed to 44.7% of the cohort. Polytherapy was highly prevalent, occurring in 70% of cases, whereas generic prescribing was observed in only 8% of prescriptions. Patients demonstrated high medication adherence, with a mean MARS-10 score of 8.1±1.7 (68.7% classified as highly adherent). ADRs were reported by 19.3% of patients, most commonly presenting as gastritis (7.3%) and insomnia or restlessness (4.7%). No severe ADRs were noted, and there were no significant subgroup differences in adherence or ADR incidence between SSRI and benzodiazepine users versus non-users (P>0.05).

Conclusions

The predominant use of SSRIs in this setting aligns with standard pharmacological management for anxiety disorders. However, the high prevalence of anxiolytic polytherapy, substantial concurrent use of benzodiazepines, and an overwhelming preference for brand-name prescriptions highlight critical areas for clinical optimization. While medication adherence is robust and medications are generally well-tolerated, prioritizing rational monotherapy, implementing planned benzodiazepine tapering, and encouraging generic prescribing could further enhance the cost-effectiveness and safety of psychiatric care.

## Introduction

Anxiety disorders encompass a spectrum of conditions characterized by excessive fear, worry, and physiological hyperarousal. They are among the most common psychiatric disorders worldwide, with the World Health Organization estimating that 301 million people (approximately 4% of the global population) were living with an anxiety disorder in 2019 [1]. In India, cross-sectional community surveys report a prevalence of approximately 2.5% to 3%, highlighting a significant public health burden [2,3]. Generalized anxiety disorder (GAD), panic disorder, phobic anxiety, obsessive-compulsive disorder (OCD), somatoform disorders, and stress-related disorders constitute the major categories of the anxiety spectrum [4], often causing significant distress and impairment in daily functioning.

Pharmacological therapy plays a central role in the management of moderate-to-severe anxiety disorders. Contemporary guidelines recommend using antidepressants - particularly selective serotonin reuptake inhibitors (SSRIs) and serotonin-norepinephrine reuptake inhibitors (SNRIs) - as the first-line long-term pharmacotherapy for chronic anxiety due to their broad efficacy and low risk of dependence [5-7]. Benzodiazepines, despite their rapid anxiolytic and hypnotic effects, carry significant risks of tolerance, dependence, cognitive impairment, and withdrawal. Consequently, their use should be restricted to short-term or intermittent applications, usually serving as adjuncts during the initial period of SSRI/SNRI treatment [5]. Alternative agents, such as buspirone, pregabalin, and β-blockers, may be considered in specific clinical scenarios [8].

Evaluating real-world prescribing patterns is critical for determining whether clinical practice aligns with evidence-based guidelines and for highlighting potential issues such as polypharmacy, a preference for brand-name drugs, and low rates of generic prescribing. In India, few recent studies have specifically examined the prescription of anti-anxiety medications. A multi-center survey by the Indian Psychiatric Society noted that SSRIs and clonazepam were the most frequently prescribed antidepressants and benzodiazepines, respectively, while generic prescribing remained notably low [9,10]. Other studies have highlighted regional variability in drug choice and the persistence of long-term benzodiazepine use despite guideline warnings to the contrary [11-13].

In addition to prescribing patterns, patient adherence and adverse drug reactions (ADRs) significantly influence treatment outcomes. Non-adherence can undermine therapeutic efficacy, while unrecognized or poorly managed side effects often lead to premature discontinuation of therapy. Currently, there is limited data on medication adherence and the incidence of ADRs among patients receiving anti-anxiety medications in India. Understanding these factors is essential for informing strategies that enhance treatment success and patient safety.

To address these gaps, this study aimed to evaluate the pharmacological management of patients with anxiety disorders in a tertiary care psychiatry outpatient setting. The baseline sociodemographic profile and diagnostic distribution of the patient cohort were determined first. The primary objective is to evaluate psychotropic prescribing patterns, including drug class distribution, combination therapy (polytherapy), and generic versus brand-name prescribing rates, whereas the secondary objectives are to assess patient-reported medication adherence using the Medication Adherence Rating Scale (MARS-10) and document the incidence, nature, and severity of ADRs and to perform exploratory subgroup analyses comparing adherence and ADR rates between drug class users and non-users (specifically SSRI users vs. non-users, and benzodiazepine users vs. non-users).

## Materials and methods

Study design and setting

This cross-sectional observational study was conducted in the psychiatry outpatient department (OPD) of a tertiary-care teaching hospital in northern India. The study was performed over a six-month period from April to September 2023. The study protocol was reviewed and approved by the Institutional Ethics Committee of Vardhman Mahavir Medical College and Safdarjung Hospital, New Delhi (Approval number: CC-254, dated: March 27, 2023). All procedures conformed to the ethical standards of the Declaration of Helsinki, and written informed consent was obtained from all participants prior to enrollment. The study and its reporting adhered to the Strengthening the Reporting of Observational Studies in Epidemiology (STROBE) guidelines.

Participants

Patients were eligible for inclusion if they were between 18 and 60 years of age, attended the psychiatry OPD during the study period, met the International Classification of Diseases, 10th Revision (ICD-10) diagnostic criteria for an anxiety spectrum disorder (codes F40-F48) as determined by the treating psychiatrist [4], and were prescribed at least one anti-anxiety medication. Anxiety spectrum disorders encompassed GAD, panic disorder, phobic anxiety disorders (including social anxiety disorder and agoraphobia), OCD, somatoform disorders, and stress-related disorders (such as post-traumatic stress disorder and adjustment disorders). Patients with psychotic disorders, bipolar affective disorder, severe cognitive impairment, or those who declined consent were excluded. Prescribing patterns for these severe mental illnesses inherently differ and typically involve antipsychotics or mood stabilizers rather than primary anxiolytics. During the study period, a total of 185 patients attending the psychiatry OPD were screened for eligibility. Of these, 20 patients were excluded (due to non-anxiety primary diagnoses or meeting exclusion criteria), and 15 patients declined participation or did not complete the evaluation, resulting in a final analyzed cohort of 150 consecutive eligible patients. The sample size was determined based on the recommendations outlined in the World Health Organization (WHO) guidelines for investigating drug use in health facilities [14]. A minimum sample size of 150 consecutive patients was targeted to adequately evaluate prescribing patterns and ADR indicators.

Data collection

A structured case record form was utilized to collect patient data. Sociodemographic variables included age, sex, educational level, occupation, domicile (urban/rural), family type (nuclear or joint), and socioeconomic status based on the modified BG Prasad scale. Clinical variables comprised the primary ICD-10 diagnosis and any comorbid psychiatric conditions.

Prescription details were comprehensively documented for each patient, including the generic and brand names of drugs, doses, frequency, and route of administration. Anti-anxiety medications were categorized into SSRIs, SNRIs, benzodiazepines, non-benzodiazepine anxiolytics (e.g., buspirone), tricyclic antidepressants, other antidepressants (e.g., mirtazapine, bupropion), anticonvulsants (e.g., pregabalin), β-blockers (e.g., propranolol), and adjunctive antipsychotics. Polytherapy was defined as the concomitant use of two or more drugs intended to alleviate anxiety symptoms (e.g., an SSRI plus a benzodiazepine, or two antidepressants). To assess generic prescribing practices, prescriptions containing at least one drug written by its generic name were noted. Prescribing metrics were calculated at two levels: (a) patient-level generic coverage, defined as the percentage of patients receiving at least one generic drug and (b) overall generic prescribing rate, calculated as the proportion of total prescribed individual drugs written by generic name relative to all prescribed medications.

Medication adherence was evaluated using the 10-item Medication Adherence Rating Scale (MARS-10) [15]. The scale comprises yes/no statements relating to medication-taking behavior and attitudes, with total scores ranging from 0 (poor adherence) to 10 (excellent adherence). For descriptive analyses, adherence was categorized as high (scores 8-10), moderate (6-7), or low (≤5).

ADRs were identified through patient interviews using a checklist of common psychotropic side effects (e.g., gastrointestinal symptoms, insomnia, loss of appetite, nausea, headaches, palpitations, constipation, sedation, and sexual dysfunction). Patients were asked to indicate whether they had experienced any of these symptoms since initiating their medication. When an ADR was reported, detailed clinical data were elicited. Causality assessment was systematically performed using the World Health Organization-Uppsala Monitoring Centre (WHO-UMC) causality assessment scale [16], while severity grading was evaluated using the modified Hartwig and Siegel Severity Assessment Scale [17].

Outcome measures

The primary outcomes evaluated were: (1) the frequency and pattern of anti-anxiety drug classes prescribed, (2) the proportion of patients receiving polytherapy versus monotherapy, and (3) the proportion of prescriptions utilizing generic names. Secondary outcomes included: (1) the distribution of adherence scores (mean±standard deviation and categories of high/moderate/low adherence) and (2) the incidence and types of ADRs. Subgroup analyses were conducted to compare mean adherence scores and overall ADR incidence between patients receiving SSRIs versus those not receiving SSRIs, as well as between those receiving benzodiazepines versus those not receiving benzodiazepines.

Statistical analysis

Data were entered into Microsoft Excel (Microsoft, Redmond, WA) and analyzed using IBM SPSS Statistics for Windows, Version 23.0 (IBM Corp., Armonk, NY). Differences between subgroups were examined using the Chi-square (χ²) test for categorical variables and the independent-samples t-test for continuous variables. A P-value of <0.05 was considered statistically significant.

## Results

Sociodemographic and clinical characteristics

During the study period, a total of 185 patients attending the psychiatry OPD were screened for eligibility. Of these, 20 patients were excluded (due to non-anxiety primary diagnoses or meeting exclusion criteria), and 15 patients declined participation, resulting in a final analyzed cohort of 150 (N=150) consecutive eligible patients. The mean age was 37.6±11.2 years (range: 18-60 years). The majority of participants were women (n=93, 62.0%). The largest age group was 31-45 years (n=66, 44.0%), followed by 18-30 years (n=45, 30.0%) and 46-60 years (n=39, 26.0%). Thirty-four percent (n=51) of patients had completed higher secondary or graduate education, 40.0% (n=60) had primary or secondary schooling, and 26.0% (n=39) had no formal education. Most participants were homemakers or unemployed (n=82, 54.7%); 25.3% (n=38) were employed in government or private jobs, and 20.0% (n=30) were self-employed or laborers. Sixty percent (n=90) of patients resided in urban areas, while 40.0% (n=60) came from rural areas.

Clinically, GAD was the most common primary diagnosis, affecting 45 patients (30.0%). Panic disorder was diagnosed in 22 patients (14.7%), and OCD was diagnosed in 20 patients (13.3%). Somatoform disorders accounted for 8.0% (n=12), stress-related disorders for 6.7% (n=10), and mixed anxiety-depressive disorder for 5.3% (n=8). Phobic anxiety disorders (including social phobia, agoraphobia, and specific phobias) accounted for 10.0% (n=15). The remaining 12.0% (n=18) comprised depressive disorders with prominent anxiety or other neurotic conditions. Table 1 displays the distribution of diagnoses.

**Table 1:** Distribution of primary diagnoses (N=150) *Includes depressive disorders with anxiety, dissociative disorders, and other unspecified neurotic conditions. ICD-10: International Classification of Diseases, 10th Revision.

Diagnosis	ICD-10 Code	Count (n)	Percentage (%)
Generalized anxiety disorder	F41.1	45	30
Panic disorder	F41.0	22	14.7
Obsessive-compulsive disorder	F42.0	20	13.3
Somatoform disorders	F45.9	12	8
Stress-related disorders	F43.9	10	6.7
Mixed anxiety-depressive disorder	F41.2	8	5.3
Social phobia	F40.1	6	4
Specific phobias	F40.2	5	3.3
Agoraphobia	F40.0	4	2.7
Other neurotic conditions*	N/A	18	12

Prescribing patterns

All participants (N=150) were prescribed at least one anti-anxiety or psychotropic medication. SSRIs were the most frequently prescribed antidepressant class. Sertraline (n=35, 23.3%) and escitalopram (n=34, 22.7%) were the most utilized SSRIs, followed by fluoxetine (n=26, 17.3%), paroxetine (n=21, 14.0%), and fluvoxamine (n=3, 2.0%). Among tricyclic antidepressants (TCAs), amitriptyline was prescribed to 35 patients (23.3%), whereas clomipramine and nortriptyline were each prescribed to two patients (1.3%). SNRIs included duloxetine (n=10, 6.7%), desvenlafaxine (n=1, 0.7%), and venlafaxine (n=1, 0.7%). Other antidepressants comprised mirtazapine (n=8, 5.3%) and bupropion (n=2, 1.3%).

Benzodiazepines were prescribed to 66 patients, with clonazepam being the most frequent (n=34, 22.7%), followed by lorazepam (n=24, 16.0%) and etizolam (n=8, 5.3%). Non-benzodiazepine anxiolytics and sedative-hypnotics included buspirone (n=1, 0.7%) and zolpidem (n=1, 0.7%). Propranolol was prescribed to 27 patients (18.0%). Among antiepileptics and gamma-aminobutyric acid (GABA) analogues, pregabalin was used in 18 patients (12.0%), while gabapentin, carbamazepine, clobazam, levetiracetam, phenytoin, and valproate were each prescribed to one patient (0.7%). Lithium carbonate was prescribed to one patient (0.7%).

Atypical antipsychotics were prescribed as augmentation therapy in 10 patients (6.7%), including aripiprazole (n=3, 2.0%), cariprazine (n=2, 1.3%), olanzapine (n=2, 1.3%), clozapine (n=1, 0.7%), quetiapine (n=1, 0.7%), and risperidone (n=1, 0.7%). Flunarizine was prescribed to one patient (0.7%).

Polytherapy of psychotropics - defined as the concurrent use of two or more agents - was observed in 105 patients (70.0%). The most common combination was an SSRI with a benzodiazepine, prescribed to 59 patients (39.3%). Eight patients (5.3%) received benzodiazepine monotherapy without an SSRI, whereas 51 patients (34.0%) received SSRI monotherapy without a benzodiazepine. The remaining 32 patients (21.3%) received other combination regimens. The median number of total psychotropic medications per prescription was 2 (interquartile range: 2-3), with a mean of 2.8±1.3. Only 12 patients (8.0%) had at least one drug prescribed by its generic name. Table 2 details the complete distribution of prescribed medications.

**Table 2:** List of anti-anxiety drugs prescribed to the participants SSRI: Selective serotonin reuptake inhibitor; SNRI: serotonin-norepinephrine reuptake inhibitor; GABA: gamma-aminobutyric acid.

Drug Class	Drug Subclass	Drug Name	Number of Patients	Percentage (%)
Antidepressants	Tricyclic Antidepressants	Amitriptyline	35	23.3
		Clomipramine	2	1.3
		Nortriptyline	2	1.3
	SSRIs	Escitalopram	34	22.7
		Fluoxetine	26	17.3
		Fluvoxamine	3	2.0
		Paroxetine	21	14.0
		Sertraline	35	23.3
	SNRIs	Desvenlafaxine	1	0.7
		Duloxetine	10	6.7
		Venlafaxine	1	0.7
	Atypical Antidepressants	Bupropion	2	1.3
		Mirtazapine	8	5.3
Anxiolytics & Sedatives	Benzodiazepines	Clonazepam	34	22.7
		Lorazepam	24	16.0
		Etizolam	8	5.3
	Non-Benzodiazepine Anxiolytics	Buspirone	1	0.7
	Sedative-Hypnotics	Zolpidem	1	0.7
Antipsychotics	Atypical Antipsychotics	Aripiprazole	3	2.0
		Cariprazine	2	1.3
		Clozapine	1	0.7
		Olanzapine	2	1.3
		Quetiapine	1	0.7
		Risperidone	1	0.7
Antiepileptics	GABA Analogues	Gabapentin	1	0.7
		Pregabalin	18	12.0
	Others	Carbamazepine	1	0.7
		Clobazam	1	0.7
		Levetiracetam	1	0.7
		Phenytoin	1	0.7
		Valproate	1	0.7
Mood Stabilizers	Lithium	Lithium Carbonate	1	0.7
Beta-Blockers	Beta-Adrenergic Blockers	Propranolol	27	18.0
Other Medications	Miscellaneous	Flunarizine	1	0.7

Adherence

The mean MARS-10 score across the sample was 8.1±1.7. High adherence (score 8-10) was observed in 103 patients (68.7%), moderate adherence (score 6-7) in 33 patients (22.0%), and low adherence (≤ 5) in 14 patients (9.3%). Adherence did not differ significantly between patients on SSRIs and those not on SSRIs (mean 8.1 vs. 8.0; t=0.43, p=0.67, independent-samples t-test) or between those on benzodiazepines and those not (mean 8.2 vs. 8.0; t=0.68, p=0.50, independent-samples t-test).

Adverse drug reactions

Twenty-nine patients (19.3%) out of 150 reported at least one adverse drug reaction (ADR). The most common ADR was gastritis or epigastric pain (n=11, 7.3%), attributed primarily to SSRI use. Seven patients (4.7%) experienced insomnia or increased restlessness. Loss of appetite occurred in four patients (2.7%); nausea in three (2.0%); restlessness/akathisia in two (1.3%); headache in one (0.7%); palpitations in one (0.7%); constipation in one (0.7%); and sexual dysfunction in one (0.7%). All reported ADRs fell into Levels 1-2 (Mild/Moderate) with zero severe events (Levels 5-7). No serious ADRs were observed. Overall ADR incidence was similar in SSRI users versus non-users (18.2% vs. 22.5%; χ²=0.35, p=0.55, Chi-square test) and in benzodiazepine users versus non-users (17.9% vs. 20.5%; χ²=0.16, p=0.70, Chi-square test). Table 3 summarizes the ADRs.

**Table 3:** Adverse drug reactions reported by patients (N=150) *Individual ADR counts sum to 31 total events because two patients reported two concurrent ADRs. ADR: Adverse drug reaction; SSRI: selective serotonin reuptake inhibitor; TCA: tricyclic antidepressant.

Adverse Drug Reaction	Associated Medications	Count (n)*	Percentage (%)*
Total patients experiencing ≥1 ADR	Various	29	19.3
Gastritis/epigastric pain	Mainly SSRIs (escitalopram, sertraline)	11	7.3
Insomnia/sleep disturbance	SSRIs (escitalopram, fluoxetine)	7	4.7
Loss of appetite	SSRIs (various)	4	2.7
Nausea	SSRIs (sertraline, fluoxetine)	3	2
Akathisia/motor restlessness	Atypical antipsychotic (aripiprazole) ± SSRI	2	1.3
Headache	SSRI (paroxetine)	1	0.7
Palpitations	SSRI (sertraline)	1	0.7
Constipation	TCA (amitriptyline)	1	0.7
Sexual dysfunction	SSRI (paroxetine)	1	0.7

## Discussion

Our cross-sectional study provides a comprehensive overview of how anti-anxiety medications are prescribed and tolerated in a tertiary-care psychiatry outpatient department in northern India. The findings reveal several important clinical patterns. First, SSRIs were the most frequently prescribed anxiolytic drugs, used by nearly three-quarters of the patients. Sertraline and escitalopram together accounted for almost half of all prescriptions. This is consistent with global and Indian guidelines recommending SSRIs as first-line pharmacotherapy for chronic anxiety disorders [6,8]. The prominence of SSRIs reflects both their proven efficacy and their improved tolerability compared with older agents. Our results also concur with national surveys indicating that escitalopram and sertraline remain the top choices among Indian psychiatrists [9].

Second, benzodiazepines were prescribed to almost 45% of patients, usually in combination with an SSRI. Clonazepam was the most utilized benzodiazepine, followed by lorazepam, which aligns with findings from other Indian studies [5]. The widespread use of benzodiazepines underscores their perceived utility in providing rapid symptomatic relief and managing insomnia or agitation during the early stages of SSRI therapy. However, benzodiazepines carry significant risks of tolerance and dependence, a challenge that has been historically documented across diverse outpatient settings [13]. Therefore, international and regional guidelines caution against their long-term use [5,6,8]. Regular clinical reviews and structured tapering schedules are essential to minimize patient harm.

Third, polytherapy was highly prevalent, with 70% of patients receiving two or more anxiolytic medications. The most frequent combination was an SSRI plus a benzodiazepine. While combination therapy can be rational during the initial treatment phase (e.g., using a benzodiazepine as a bridging agent until the SSRI takes effect), prolonged polypharmacy increases the risk of drug-drug interactions and adverse effects without offering clear long-term benefits. Our observed polytherapy rate appears higher than those reported in some earlier Indian studies [11]. This variance may reflect differences in definitions (we defined SSRI plus a benzodiazepine as polytherapy) or patient populations, as our tertiary center may manage more severe or treatment-refractory cases. Nevertheless, clinicians should routinely aim to rationalize therapeutic regimens to the minimum effective number of drugs.

Fourth, only 8% of prescriptions included a generic drug name, indicating an overwhelming reliance on brand-name medications. This pattern echoes previous Indian reports [11] and highlights a significant area for systemic improvement. Generic prescribing significantly reduces out-of-pocket costs and can consequently improve medication accessibility and long-term adherence. Encouraging prescribers to use generic names where available, alongside ensuring strict quality assurance of generic psychotropics, could greatly enhance rational pharmacotherapy.

Medication adherence within the cohort was generally favorable. The mean MARS-10 score was 8.1, with over two-thirds of patients classified as highly adherent. This rate is better than adherence levels often reported for other psychiatric disorders, possibly because anxiety symptoms are highly distressing, and patients rapidly appreciate the symptomatic relief provided by treatment. High adherence may also reflect the supportive, psychoeducational environment of a tertiary care center and the relatively short duration of therapy for many participants. Importantly, adherence did not differ significantly between patients on SSRIs and those on benzodiazepines, or between those on polytherapy versus monotherapy, suggesting that the prescription of multiple drugs did not inherently compromise compliance. However, roughly one-third of patients still demonstrated moderate or low adherence - often resulting from the self-discontinuation of medication upon symptom improvement or due to a fear of side effects. Routine assessment of adherence during follow-up visits, targeted patient education, and caregiver involvement are necessary to further optimize adherence.

The incidence of ADRs was modest (19.3%), and the majority of reported reactions were mild. Gastrointestinal complaints (gastritis, nausea) and insomnia were the most common, which is consistent with the well-documented side-effect profiles of SSRIs. No serious ADRs were observed. Notably, no patients spontaneously reported daytime sedation or cognitive dulling despite the widespread use of benzodiazepines; this may indicate that sedation was either not perceived as an undesirable effect by the patients or was simply under-reported. Sexual dysfunction, a notoriously common SSRI side effect, was reported by only one male patient, strongly suggesting under-reporting likely driven by cultural stigma. Clinicians must proactively inquire about such sensitive side effects and offer management strategies, such as dose adjustments, drug holidays, or switching therapeutic agents. Furthermore, there was no significant difference in the overall ADR incidence between SSRI and non-SSRI users, or between benzodiazepine and non-benzodiazepine users, indicating comparable short-term tolerability profiles.

These findings should be interpreted in the context of several limitations. This was a single-center study with a relatively small sample size and a cross-sectional design; therefore, the results may not generalize to primary care settings or other geographic regions of India. The reliance on patient self-reporting for adherence and ADRs introduces the potential for recall and social desirability biases, as objective measures were not feasible. Additionally, our cross-sectional design provides only a single snapshot of patient care. Because specific treatment durations were not formally quantified, our ability to evaluate delayed ADRs, longitudinal adherence, and long-term benzodiazepine dependence is inherently limited. All subgroup analyses (SSRI users vs. non-users and benzodiazepine users vs. non-users) were exploratory and unadjusted for potential confounders such as specific psychiatric diagnosis, disease severity, or polytherapy status. Additionally, our study did not assess the concurrent use of complementary and alternative medicines (CAMs). Unmeasured CAM use represents a potential confounder, as evaluating its inclusion could have provided a more comprehensive picture of real-world treatment patterns, unexpected drug-drug interactions, non-reported adverse effects, and overall medication adherence. Furthermore, because no formal prior power calculation was performed for these subgroup comparisons, statistically non-significant findings must be interpreted with caution as potentially underpowered rather than conclusive evidence of equivalence. Future prospective studies collecting data at standardized follow-up intervals are necessary to properly assess these long-term outcomes. The study also grouped diverse anxiety-related disorders together, though ideal prescribing patterns might differ across specific diagnostic subtypes. Finally, we did not evaluate clinical outcomes (e.g., standardized symptom reduction, functional improvement) or the concurrent use of non-pharmacological interventions like psychotherapy, which can significantly influence both medication requirements and adherence. Future longitudinal, multicenter studies with larger sample sizes are warranted to validate these findings and explore long-term clinical outcomes.

Despite these limitations, our study provides valuable real-world insights into contemporary prescribing practices for anxiety disorders within a major Indian tertiary-care hospital. The results suggest that while clinicians largely follow guideline-recommended practices by utilizing SSRIs as the foundation of treatment, they also rely heavily on benzodiazepines for symptomatic management. The high rates of patient adherence and the low incidence of severe ADRs are reassuring. However, the high prevalence of polytherapy and the strong preference for brand-name prescribing identify clear opportunities for clinical optimization. Strengthening adherence monitoring, ensuring the timely and appropriate tapering of benzodiazepines, and actively promoting generic prescribing are critical steps toward enhancing the cost-effectiveness and safety of anxiety disorder management.

## Conclusions

In this tertiary outpatient anxiety cohort, psychotropic prescribing strongly aligned with guidelines through preferential SSRI use; however, high rates of benzodiazepine co-prescribing, polytherapy, and low generic adoption signal key areas for quality and cost optimization. Patient adherence (MARS-10) was predominantly moderate-to-high, and all adverse drug reactions were mild-to-moderate (Hartwig Levels 1-2), with zero severe or life-threatening events. Exploratory subgroup analyses showed no significant differences in adherence or ADRs between drug class users and non-users (SSRIs and benzodiazepines), though these findings should be interpreted cautiously due to power limitations. Clinical care can be enhanced by promoting generic prescribing, implementing structured adherence counseling, and enforcing planned benzodiazepine tapering.
